# Evaluation of the cytotoxic activity of chemically characterized propolis originating from different geographic regions and vitamin D co-supplementation against human ovarian cancer cells

**DOI:** 10.1186/s13048-024-01500-6

**Published:** 2024-09-07

**Authors:** Eman Ali, Maged W. Helmy, Eman H. Radwan, Karoline K. Abdul Aziz, Aida A. Abd El-Wahed, Lamia M. El-Samad, Abeer El Wakil

**Affiliations:** 1 Department of Zoology, Faculty of Science, Damanhour University, Damanhour, Egypt; 2 Department of Pharmacology and Toxicology, Faculty of Pharmacy, Damanhour University, Damanhour, Egypt; 3 Department of Pharmacology and Toxicology, College of Pharmacy, Arab Academy for Science, Technology and Maritime Transport, Alexandria, Egypt; 4 Department of Bee Research, Plant Protection Research Institute, Agricultural Research Centre, Giza, 12627 Egypt; 5 Department of Zoology, Faculty of Science, Alexandria University, Alexandria, Egypt; 6 Department of Biological and Geological Sciences, Faculty of Education, Alexandria University, Alexandria, 21526 Egypt

**Keywords:** Propolis, LC-MS/MS, Oxidative stress, Toxicity, OVCAR4

## Abstract

Ovarian cancer is the second most common and lethal gynecologic malignancy. Among natural product-based therapy, the honeybee products, particularly propolis, serve a valuable source contributing directly to human nutrition and health.

In the present study, we determined the chemical composition of different types of propolis originating from Egypt, Germany and France using liquid chromatography-tandem mass spectrometry. The compounds identified belong to different metabolite classes, including flavonoids, cinnamic acid, chalcones, terpenoids, phenolic lipids, stilbenes, phenolic compounds, carbohydrates, vitamins, coumarins, polyprenylated benzophenone, benzoic acids, fatty acid methyl ester, and coumaric acid, and their derivatives. The most active extract is from France then Egypt and Germany.

Afterwards, we treated the human ovarian cancer cells, OVCAR4, with different concentrations (1–400 μg/mL) of variable propolis types supplemented or not with vitamin D (0.0015–0.15 μg/mL) in order to evaluate the efficacy and the cytotoxic activities of our local P as compared to other types collected from different geographic regions. Importantly, the combinatorial treatment of OVCAR4 cancer cells with propolis and vitamin D in the same concentration ranges resulted in enhanced cell viability inhibition. Furthermore, such co-supplementation with vitamin D inhibits predominately the proliferative activity of cell population with the French propolis type as manifested by Ki67 expression, while it reduces considerably its expression, particularly with the German type, followed by the Egyptian one.

Nowadays, scientists are interested by natural products which have risen to the forefront of drug discovery. Chemically characterized propolis showing cell viability inhibition and antiproliferative potential seems a valuable extract for further consideration as anti-carcinogenic agent.

**Graphical abstract:**

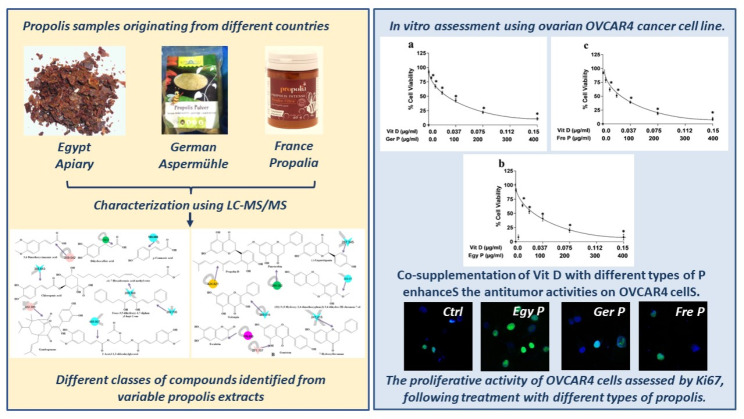

**Supplementary Information:**

The online version contains supplementary material available at 10.1186/s13048-024-01500-6.

## Introduction

Non-communicable diseases (NCDs), including among others cardiovascular diseases, chronic respiratory diseases, cancers, diabetes, urogenital, blood and endocrine diseases, are collectively the leading causes of death and disability in the world. The Global Burden of Diseases (GBD), Injuries, and Risk Factors Study 2019 provides the most up-to-date assessment of a mutually exclusive and collectively exhaustive list of diseases and injuries for 204 countries and territories from 1990 to 2019 [1, 2]. Morbidity and mortality caused by cancer are mainly due to changes in exposure to risk factors. It has been reported that more deaths worldwide are caused by cancer than by cardiovascular diseases [3]. The cancerous process is a result of disturbed cell function due to the accumulation of many genetic and epigenetic aberrations [4, 5]. It is difficult to assess the validity of individual aetiological factors, but it can be concluded that interaction of various risk factors has the largest contribution to the cancer development. Environmental, exogenous and endogenous factors, as well as individual factors including genetic predisposition, contribute to the development of cancer [6, 7].

Ovarian cancer is the second most common and lethal gynecologic malignancy. So far, there is lack of methods recommended for screening and early diagnosis of this disease [8]. Nowadays, scientists are interested in developing naturally-derived drugs [9, 10], particularly that the nature has been the source of life-changing and saving medications for centuries. Plant-derived anticancer constituents including vinblastine, vincristine, paclitaxel, curcumin, colchicine, and lycopene are examples of nature’s gifts to medicine [11]. Among the different honeybee products, propolis (P) serves a valuable source contributing directly to human nutrition and health [12]. The chemical composition of P is diverse and depends on the geographical and botanical origin, i.e., climate factors, plant resources, place of origin, and time in which it was collected by the bees. The specificity of the local flora is the main factor that determines the chemical composition of P and, subsequently, its biological and pharmacological properties [13]. In general, propolis is composed of 50–60% of resins and balms, 30–40% of waxes and fatty acids, 5–10% of essential and aromatic oils, 5–10% of pollen, and about 5% of other substances, such as amino acids, vitamins, macro-, and microelements [14]. Vitamin D which is produced endogenously in the skin by a photochemical reaction is the precursor of 1,25-dihydroxyvitamin D in the organism, a steroid hormone involved in various vital processes in the body, including pathways that inhibit cancer promotion and progression. It has received wide scientific interest in cancer prevention research and cancer therapy as well [15, 16]. Moreover, a bulk of research indicates that low levels of circulating vitamin D are linked to an increased risk of developing cancer, whereas supplementation may further enhance clinical outcomes. These encouraging results nevertheless need additional study and development of cutting-edge strategies that target vitamin D signalling and metabolic systems to enhance cancer therapeutic outcomes [17].

In the present study, we determined the chemical composition of different types of P originating from Egypt (Egy P), Germany (Ger P), and France (Fre P) using liquid chromatography-tandem mass spectrometry (LC-MS/MS). Afterwards, we investigated the in vitro antiproliferative potential of the different types of P against human ovarian cancer cell line supplemented or not with vitamin D in order to mimic the internal environment within the body and maintain a relatively similar biological condition to evaluate the efficacy and the cytotoxic activities of our local P as compared to other types collected from different geographic regions. Our study combined biochemical approaches and molecular biology techniques in order to elucidate the cellular and molecular effects of P against OVCAR4 cancer cells.

## Results

### Identification of the secondary metabolites from the different types of P

**Fig. 1:**
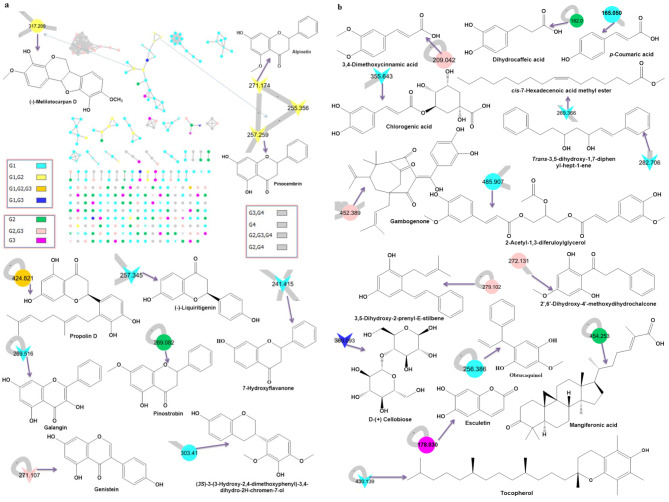
Chemical structure of (**a**) Flavonoids compounds, and (**b**) Different classes of compounds identified from different propolis extracts using LC–LTQ–MS/MS analysis and assisted Global Natural Product Social (GNPS) molecular networking. The network is represented as a pie chart, where nodes correspond to parent masses of the metabolites. Circular nodes represent unique detected peaks in the molecular networking, while triangle nodes denote parent ions identified in the GNPS molecular networking. Grey nodes are the metabolites detected in the blank solvent and those common between propolis samples and the blank. The metabolites in propolis from Egypt, Germany, and France are represented by aqua, green, and violet colors, respectively. Yellow nodes indicate metabolites that occur in Egyptian and German propolis, while orange nodes denote metabolites common across all three regions’ propolis samples. Blue nodes represent similar metabolites between Egyptian and French propolis, and pink nodes indicate metabolites common between German and French propolis. G1: Propolis from Egypt, G2: Propolis from Germany, G3: Propolis from France, and G4: Blank solvent

The three different propolis extracts from Egypt, Germany, and France each had a unique metabolomics mass profile that was analyzed using a Global Natural Products Social Molecular Networking (GNPS) network based on tandem mass spectrometry data (Fig. 1; Table 1) in the positive ionization mode [15, 18, 19]. The molecular network’s nodes represented the metabolites, and chemically similar metabolites were grouped together [20]. The parent ions of propolis were assigned 249 colored nodes. The LC-MS/MS analysis of propolis extracts revealed the presence of a complex mixture, and the chemical components of the extracts identified are summarized in Table 1. 57 compounds were identified; 9 parent ions matched nine known standards in the GNP library (Table 1), belonging to flavonoids, cinnamic acids, carbohydrates, vitamins, and fatty acid methyl ester. Pinocembrin, (-)-liquiritigenin, and chlorogenic acid were previously identified from propolis and matched with the GNPS database. 49 compounds were identified previously in propolis as shown in Table 1. The compounds identified belong to different metabolite classes, including flavonoids, cinnamic acid, chalcones, terpenoids, phenolic lipids, stilbenes, phenolic compounds, carbohydrates, vitamins, coumarins, polyprenylated benzophenone, benzoic acids, fatty acid methyl ester, and coumaric acid and their derivatives.

**Table 1:** Identification of compounds from propolis originating from different regions using LC–LTQ–MS/MS analysis

Compound name	R_t_	m/z	MF	MS^2^	Egy P	Ger P	Fre P	Reference
**Flavonoid**								
5-Hydroxy-4"-4"-dimethyl-5"-methyl-5"-H-dihrofurano(2",3"6,6) flavanone	0.90	326.26	C_19_H_18_O_5_	309.2058, 295.0773, 263.2461, 238.5674, 161.1475	**×**	**-**	**-**	[21]
5,7,3’,4’Tetrahydroxy-6*C*geranylflavanone	3.02	424.03	C_25_H_28_O_6_	407.3767, 390.6158, 348.2471, 321.1401, 314.1860, 301.1189, 287.1748, 271.0090, 252.9539, 177.9554, 122.8480	**-**	**×**	**-**	[ 21]
Propolin D	3.17	424.82	C_25_H_28_O_6_	407.2590, 365.1900	**×**	**×**	**×**	[22]
Genistein	4.00	271.11	C_15_H_10_O_5_	270.9819, 253.0970, 243.0889, 215.2456, 168.7328, 152.9690, 106.9171, 99. 9084, 90.9551	**-**	**×**	**×**	[23]
(2*R*,3*R*)-6-[1-(4-Hydroxy-3-methoxyphenyl)prop-2-en-1-yl]pinobanksin	4.08	420.26	C_25_H_23_O_7_	420.0123, 406.0773, 391.1183, 375.2168, 385.1605, 257.1549, 240.1076, 137.0843, 121.0632	**-**	**×**	**-**	[24]
Pinocembrin	5.13	257.26	C_15_H_12_O_4_	229.0510, 215.0090, 211.0410, 172.9770, 152.9330, 102.9960	**×**	**×**	**-**	[25, 26] https://bit.ly/3PyvfAO *(Access on December 22, 2022)*
Daidzein	5.20	254.06	C_15_H_10_O_4_	254.9752, 237.1179, 227.0928, 219.1870, 199.0260, 186.9690, 181.2539, 147.0320, 130.0750	**-**	**×**	**-**	[27]
Hesperitin5,7dimethyl ether	5.34	330.11	C_18_H_18_O_6_	313.2244, 298.3091, 282.0153, 267.0806, 251.3294, 236.1053, 220.2451, 179.9206	**-**	**×**	**-**	[21]
(-)-Liquiritigenin	5.51	257.35	C_15_H_12_O_4_	229.0760, 210.9960, 162.06029860, 146.9370,136.9260,119.0250	**×**	**-**	**-**	[28] https://bit.ly/3FGNijs *(Access on December 22, 2022)*
Pinostrobin	6.51	269.08	C_16_H_14_O_4_	222.9600, 173.0543, 166.9160, 104.9250, 90.9250	**-**	**×**	**-**	[23]
3’,5Dihydroxy-4’,7dimenthoxy flavones	7.21	492.49	C_27_H_24_O_9_	474.2940, 330.2830, 312.2600	**×**	**-**	**-**	[21]
Abyssinoflavanone VII	8.53	424.49	C_25_H_28_O_6_	365.2060, 3271710, 281.1140, 150.9727	**-**	**×**	**×**	[29]
3’,4’-Di-*O*-benzyl-7-*O*-(2-hydroxyethyl)-3*-O*-methylquercetin	9.38	540.18	C_32_H_28_O_8_	523.2446, 479.3914, 371.2449, 327.1682, 231.1650	**-**	**-**	**×**	[23]
Alpinetin	10.34	271.17	C_16_H1_4_O_4_	229.0270, 225.04420, 203.0910, 166.9390, 152.8913, 130.9920, 103.0320	**×**	**×**	**-**	https://bit.ly/3FznPZg * (Access on December 22, 2022)*
Galangin	10.40	270.36.51	C_15_H_10_O_5_	241.8558, 197.0250, 175.9750, 167.0419, 152.9669,131.0289	**×**	**-**	**-**	[30]
2’-Hydroxyformononetin	10.80	270.24	C_15_H_10_O_5_	241.1118, 237.9930, 214.0370, 182.0510, 162.9420, 136.9990	**×**	**-**	**-**	[31]
Medicarpin	10.86	270.28	C_16_H_14_O_4_	255.0075, 243.0520, 215.0270, 164.9730, 160.9770, 136.9680, 122.9750, 109.0223	**×**	**-**	**-**	[23]
7-Hydroxy-8-methoxyflavanone	10.87	270.28	C_16_H_14_O_4_	236.9930, 213.0420, 193.0170, 166.9664,118.000	**×**	**-**	**-**	[26]
7-Hydroxyflavanone	11.04	241.42	C_15_H_12_O_3_ :	213.0680, 194.9880, 162.9450, 136.9680	**×**	**-**	**-**	https://bit.ly/3Hkdvaa * (Access on December 22, 2022)*
(-)-Melilotocarpan D	11.23	317.21	C_17_H_16_O_6_	301.9946, 299.1074, 285.0340, 178.9790, 162.9990, 152.9891, 138.9329, 134.9960	**×**	**×**	**-**	[32]
Schweinfurthin A	11.25	548.31	C_34_H_44_O_6_	549.6781, 518.8340, 506.3636, 492.3170, 478.5146, 465.4971, 437.2702, 414.8420, 394.1100, 377.7569, 299.3496, 209.9933	**-**	**×**	**-**	[21]
(*2R*,*3R*)-pinobanksin 3-(2-methyl)-butyrate	11.44	342.39	C_20_H_22_O_5_	287.0210, 268.9990, 203.0690, 164.4538,	**×**	**-**	**-**	[33]
(-)-Mucronulatol	12.62	303.41	C_17_H_18_O_5_	285.0850, 271.0620, 253.9700, 180.0468, 162.9400, 134.9500	**×**	**-**	**-**	[32]
Kaempferide	13.43	300.31	C_17_H_16_O_5_	283.0220, 268.9840, 229.1165, 152.9700, 165.9284, 136.9850, 121.0080	**×**	**-**	**-**	[30]
Rhamnocitrin	13.50	299.54	C_16_H_12_O_6_	299.2303, 271.1616, 209.1653, 165.9840, 137.0400, 122.9660	**×**	**-**	**-**	[26]
6-(1,1-dimethyl allyl) pinocembrin	15.15	324.85	C_20_H_20_O_4_	307.2065, 293.2345, 265.2312, 221.2634, 214.1630, 95.0537	**×**	**-**	**-**	[21]
**Cinnamic acid and its derivatives**								
Dihydrocaffeic acid	0.53	182.00	C_9_H_10_O_4_	136.9830, 123.8996, 107.9610	**-**	**×**	**-**	[34]
3,4-Dimethoxycinnamic acid	0.76	209.04	C_11_H_12_O_4_	191.1121, 162.9759, 133.0046, 102.9836, 76.9730	**-**	**×**	**×**	[35]
Cinnamyl caffeate	1.35	296.14	C_18_H_16_O_4_	279.0237, 238.2185, 193.0850, 162.96600, 148.97	**-**	**×**	**-**	[36]
Artepillin C (3,5-Diprenyl-4-hydroxycinnamic acid)	1.63	300.17	C_19_H_24_O_3_	300.1414, 283.2649, 255.0471, 199.9463, 145.0778	**-**	**-**	**×**	[37]
Caffeic acid	2.72	180.04	C_9_H_8_O_4_	162.9987, 147.9744, 135.0150, 121.9347	**-**	**-**	**×**	[38]
(2*E*)-3-[7-(3-methyl-2-buten-1-yl)-2-(1-methylethenyl)- 5-benzofuranyl]-2-propenoic acid	2.89	296.68	C_19_H_20_O_3_	279.0405, 222.9681, 194.9552, 151.1024,	**-**	**-**	**×**	[39]
3-Prenyl-4-methoxy cinnamic acid	2,92	246.13	C_15_H_18_O_3_	229.0949, 201.0918, 189.0085, 145.0113, 132.8638, 118.7485, 103.9059	**-**	**-**	**×**	[40]
Caffeoyl coumaroyl acetyl glycerol	3.16	442.18	C_23_H_22_O_9_	425.2443, 348.4769, 336.9940, 323.1130, 309.0520, 295.1140, 279.1529, 264.2130, 236.1262, 220.0712, 205.0080, 163.1148, 135.9840	**-**	**×**	**-**	[30]
Dimethoxycinnamic acid	3.53	208.07	C_11_H_12_O_4_	162.9500, 148.9932, 132.9710, 112.9920, 104.9582, 90.9847	**-**	**×**	**-**	[35]
(2*E*)-3-(2,2-dimethyl-2H-1-benzopyran- 6-yl)-2-propenoic acid	6.6	230.09	C_14_H_14_O_3_	213.1382, 196.9871, 185.0270, 84.9310	**-**	**×**	**-**	[39]
Chlorogenic acid	6.95	355.14	C_16_H_18_O_9_	319.0590, 309.0720, 162.9400, 135.0260	**×**	**-**	**-**	[41] https://bit.ly/3uEPsLr *(Access on December 22, 2022)*
*p*-Coumaric acid	11.58	165.05	C_9_H_8_O_3_	163.6393, 149.0293, 132.0347, 120.9949	**×**	**-**	**-**	[28]
**Chalcones**								
2',6'-Dihydroxy-4'-methoxydihydrochalcone	5.20	272.13100	C_16_H_16_O_4_	255.0665, 224.2262, 195.02 49.0642, 167.9810, 132.9940, 91.0223	**-**	**-**	**×**	[23]
(*E*,*E*,*E*)-4,2’,4’-Trihydroxy-3’-(7’’-hydroxy-3’’,7’’-dimethyloct-2’’,5’’-dienyl)-chalcone	9.51	408.19	C_25_H_28_O_5_	407.4463, 391.2099, 349.2774, 335.7463, 322.8995, 309.0037, 294.1888, 282.1763	**-**	**-**	**×**	[42]
**Terpenoids**								
Acetylisocupressic acid	3.37	362.25	C_22_H_34_O_4_	362.2958, 345.1349, 330.2925, 317.1249, 302.1115, 288.0924, 274.1560, 247.1904, 149.0241	**-**	**-**	**×**	[43]
Mangiferonic acid	6.25	454.25	C_30_H_50_O	437.2480, 422.1044, 407.0734, 394.3219, 297.1874, 215.1196	**-**	**×**	**-**	[44]
Poilaneic acid	12.68	302.46	C_20_H_30_O_2_	286.0009, 269.1046,, 255.0537, 241.0055, 227.0801, 122.9624,	**×**	**-**	**-**	[45]
**Phenolic lipids**								
5-(12’*Z*-Heptadecenyl)-resorcinol	4.96	346.29	C_23_H_38_O_2_	329.1013, 311.2385, 303.0174, 297.0928, 283.1714, 269.1329, 237.2850, 195.0435, 167.9992, 111.9131	**-**	**-**	**×**	[44]
**Stilbenes**								
3,5-Dihydroxy-2-prenyl-*E*-stilbene	5.89	279.10	C_19_H_20_O_2_	250.9272, 237.8688, 225.1041, 120.9799	-	×	×	[21]
5,4’-Dihydroxy-3,3’-dimethoxy-2-prenyl-E-stilbene	12.37	340.42	C_21_H_24_O_4_	299.0710, 284.9870, 175.0995, 104.8999	×	-	-	[46]
**Phenolic compounds**								
Obtusaquinol	11.65	255.64	C_16_H_16_O_3_	256.0338, 240.0186, 223.1327, 150.9319, 135.0651	×	-	-	[31]
(*Z*)-1-(2’-methoxy-4’,5’- dihydroxyphenyl)-2-(3-phenyl)propene	11.67	256.39		256.0931, 241.0217, 225.1016, 209.0823, 179.0548, 152.9436, 139.9608	×	-	-	[32]
*Trans*-3,5-dihydroxy-1,7-diphenyl-hept-1-ene	16.77	282.71	C_19_H_22_O_2_	264.5700, 248.2991, 191.510, 178.8278, 162.0833, 149.0860, 135163.1070, 94.9480	×	-	-	[21]
**Carbohydrates**								
Glycan 4.*β*.-Galactobiose	1.09	365.225		347.1100, 305.1200, 275.0000, 245.0790, 203.0340, 185.0210	**×**	**-**	**-**	https://bit.ly/3iUzk69 * (Access on December 22, 2022)*
D-(+) Cellobiose	12.21	342.12	C_12_H_22_O_11_	342.0950, 325.0200, 307.0130, 289.0140, 259.0080, 217.9598, 203.8954, 198.0400, 162.9530, 126.9540, 108.9790	**×**	**-**	**×**	https://bit.ly/3j8NRef * (Access on December 22, 2022)*
**Vitamin**								
Tocopherol	16.01	430.14	C_29_H_50_O_2_	401.3220, 219.1030, 205.0360, 191.0640, 177.0509, 165.0060	**×**	**-**	**-**	https://bit.ly/3uClwzA * (Access on December 22, 2022))*
**Coumarin**								
Esculetin	2.77	178.03	C_9_H_6_O_4_	149.9676, 133.0174, 122.9413,104.9516	**-**	**-**	**×**	[47]
Gambogenone	4.71	452.55	C_27_H_32_O_6_	435.3546,417.1204, 326.6595, 295.0794	**-**	**×**	**×**	[21]
**Benzoic acid**								
4-Methoxybenzoic acid	14.93	153.06	C_8_H_8_O_3_	153.0307, 134.9597, 91.9711, 107.87, 89.9705, 67.9884, 62.8947	**-**	**-**	**×**	[48]
**Fatty acid methyl ester**								
*cis*-7-Hexadecenoic acid methyl ester	15.04	269.37	C_17_H_32_O_2_	237.1210, 219.1780, 199.1270, 185.1370, 163.0620, 157.0760, 109.0590	**×**	**-**	**-**	https://bit.ly/3V63IYh * (Access on December 22, 2022)*
**Coumaric acid and its derivatives**								
2-Acetyl-1,3-diferuloylglycerol	14.53	485.91	C_25_H_26_O_10_	467.3230, 321.2061892, 309.1245	**×**	**-**	**-**	[25]

Flavonoids are the most predominant class (Table 1). The abundance of the class was in the elution range (R_t_ 1–15 min). Based on Tables 1, 26 compounds have been identified, the LC-MS revealed that propolin D was detected in Egy P, Ger P, and Fre P. Pinocembrin, alpinetin, and (-)-melilotocarpan D were detected in Egy P and Ger P. Furthermore, genistein, abyssinoflavanone VII, and gambogenone were detected in Ger P and Fre P.

Cinnamic acid and its derivatives, 3,5-diprenyl-4-hydroxycinnamic acid, caffeic acid, (2*E*)-3-[7-(3-methyl-2-buten-1-yl)-2-(1-methylethenyl)-5-benzofuranyl]-2-propenoic acid, and 3-prenyl-4-methoxy cinnamic acid were detected in Fre P while chlorogenic acid and *p*-coumaric acid characterized the Egy P. Dihydrocaffeic acid, cinnamyl caffeate, caffeoyl coumaroyl acetyl glycerol, dimethoxycinnamic acid, and (2*E*)-3-(2,2-dimethyl-2 H-1-benzopyran-6-yl)-2-propenoic acid were determined in Ger P. Acetylisocupressic acid and belong to terpenoids and were detected in Fre P. Mangiferonic acid and poilaneic acid were identified in Ger P and Egy P, respectively. 3,5-dihydroxy-2-prenyl-E-stilbene and Gambogenone were detected in both Ger P and Fre P.

### P affected ovarian cell viability

**Fig. 2:**
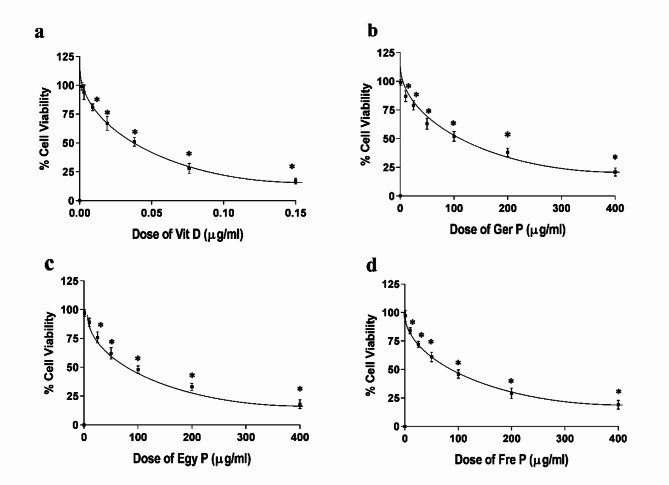
The viability of OVCAR4 ovarian cancer cells treated with different concentrations of Vit D (0.0015–0.15 μg/mL) (**a**), or with different concentrations of Propolis (1–400 μg/mL) from different geographical sources Ger P (**b**), Egy P (**c**) and Fre P (**d**), data points represent the mean ± SEM (standard error of mean), each performed in triplicate. *p < 0.05 indicates a significant difference for different types of P and Vit D vs. the corresponding control group

**Fig. 3:**
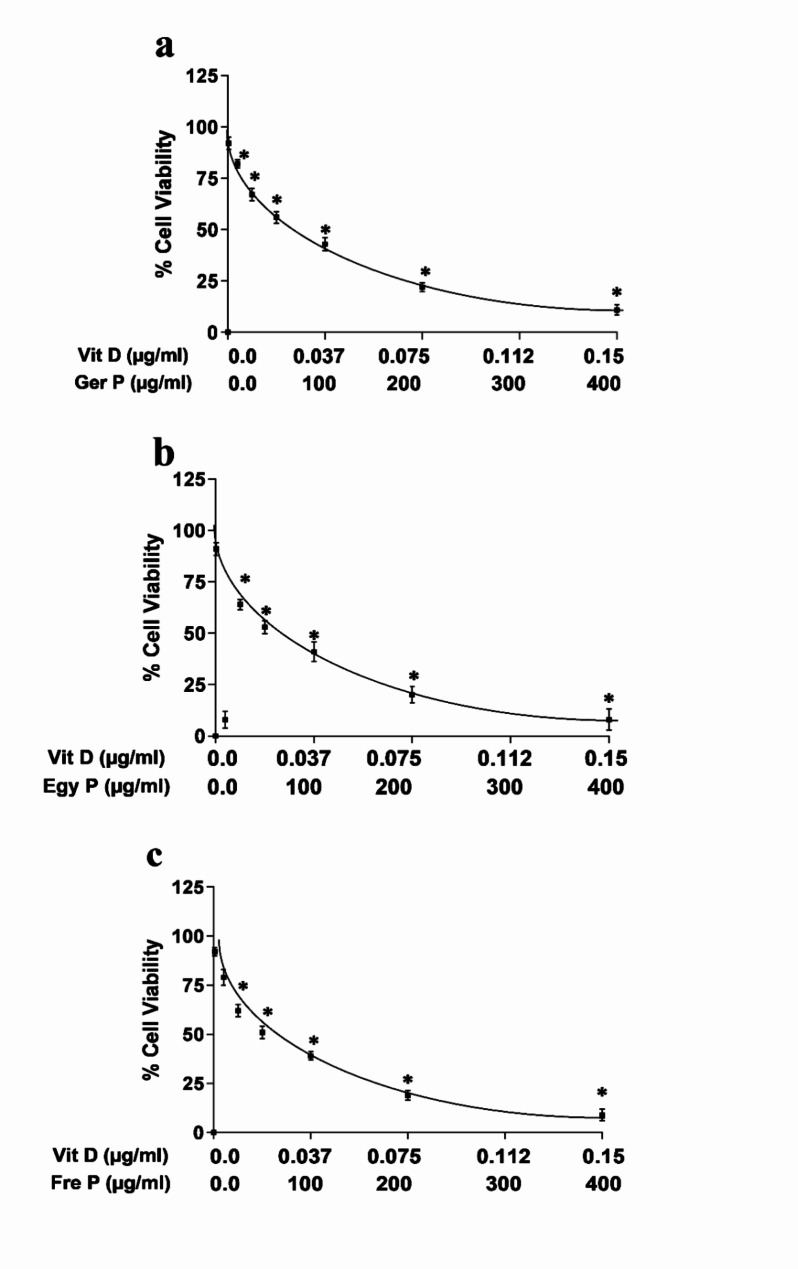
The viability of OVCAR4 ovarian cancer cells treated with combination of different concentrations of Vit D (0.0015–0.15 μg/mL) and with different concentrations of Propolis (1–400 μg/mL) from different geographical sources Ger P (**a**), Egy P (**b**) and Fre P (**c**), Data points represent the mean ± SEM (standard error of mean), each performed in triplicate. *p < 0.05 indicates a significant difference for different types of P and Vit D vs. the corresponding control group

The effect of P and/or Vit D on the growth of OVCAR4 ovarian cancer cells is presented in Fig. 2 MTT. Vit D at different concentrations (from 0.0015 to 0.15 μg/mL) produced marked growth inhibition with an IC_50_ of 0.035 ± 0.002 μg/mL (Fig. 2a). It exhibited a concentration-dependent cytotoxic effect, where exposure to the P concentration range (1–400 μg/mL) inhibited cell viability with an IC_50_s of 100.335 ± 1.38 μg/mL and 86.064 ± 2.09 μg/mL for Ger P and Egy P, respectively (Fig. 2b, c). While Fre P exerted the lowest IC_50_ equivalent to 75.040 ± 1.45 μg/mL among all other propolis (Fig. 2d). Additionally, the combination of P (from different geographical sources) and Vit D in the same concentration ranges resulted in enhanced cell viability inhibition with a combined IC_50_ values of 47.4701 ± 1.27 (47.4519 μg/mL for Ger *P* + 0.01815 μg/mL for Vit D; Fig. 3a), 38.7193 ± 1.79 (38.7045 μg/mL for Egy *P* + 0.01480 μg/mL for Vit D; Fig. 3b) and 37.0036 ± 1.41 (36.9895 μg/mL for Fre *P* + 0.01415 μg/mL for Vit D; Fig. 3c).

### Combination index (CI) and dose reduction index (DRI) of different types of P and Vit D

To examine the combined effects of different types of P and Vit D on OVCAR4 ovarian cancer cells, synergy experiments were performed. These cells were treated experimentally with different geographical sources of P, Vit D, or combination of both drugs, and CompuSyn software was used to determine the type of drug interaction between the agents. Table 2 presents the CIs detected using CompuSyn software after treatment of OVCAR4 cells with different combinations of the different types of P and Vit D. CIs values at IC_50_ were 0.984, 0.866 and 0.891, for Ger *P* + Vit D, Egy *P* + Vit D and Fre *P* + Vit D, respectively. These results demonstrate a CI value < 1 at IC_50_, revealing a synergistic relationship between the different types of P and Vit D at IC_50_ levels in the OVCAR4 cell line (Table 2). Additionally, Table 2 shows that at IC_50_ (50% inhibition achieved by the combination), the concentration of the different types of P and Vit D in their combinations were reduced by around two folds than their IC_50_ of each drugs alone, as depicted with the relevant DRI values. These observations support the hypothesis that co-supplementation of Vit D with different types of P, enhance the antitumor activities on OVCAR4 ovarian cancer cell line.

**Table 2:** CIs (combination indices) (eq. 1) and DRIs obtained using CompuSyn software to analyse OVCAR4 ovarian cell viability inhibition resulting from treatment with the combination of vit D (0.0015–0.153 μg/mL) and different geographical sources of P (1–8400 μg/mL) for 48 h

At the level of Effective dose that induce 50% cellular viability inhibition							
	CI value	Concentration of each drug alone (μM)		Concentration of each drug in combination (μM)		DRI Vit D	DRI of corresponding *P*
		IC50 of Vit D (μg/mL)	IC50 of selected geographical species of *P* (μg/mL)	IC50 of Vit D (μg/mL)	IC50 of selected geographical species of *P* (μg/mL)		
**Ger P + Vit D**	0.984	0.035	100.355	0.018	47.451	2.11	1.9
**Egy P + Vit D**	0.866	0.035	86.064	0.014	38.704	2.39	2.22
**Fre P + Vit D**	0.891	0.035	75.040	0.014	36.989	2.50	2.02

**Equation 1, combination index**^32^: CI = E (ca.) E (da) + E (cb) E (db)

Where; CI = combination index

E (ca.) = effect for drug a in combination

E (cb) = effect for drug b in combination

E (da) = effect of drug a alone

E (db) = effect of drug b alone

### P affected cell proliferation

**Fig. 4:**
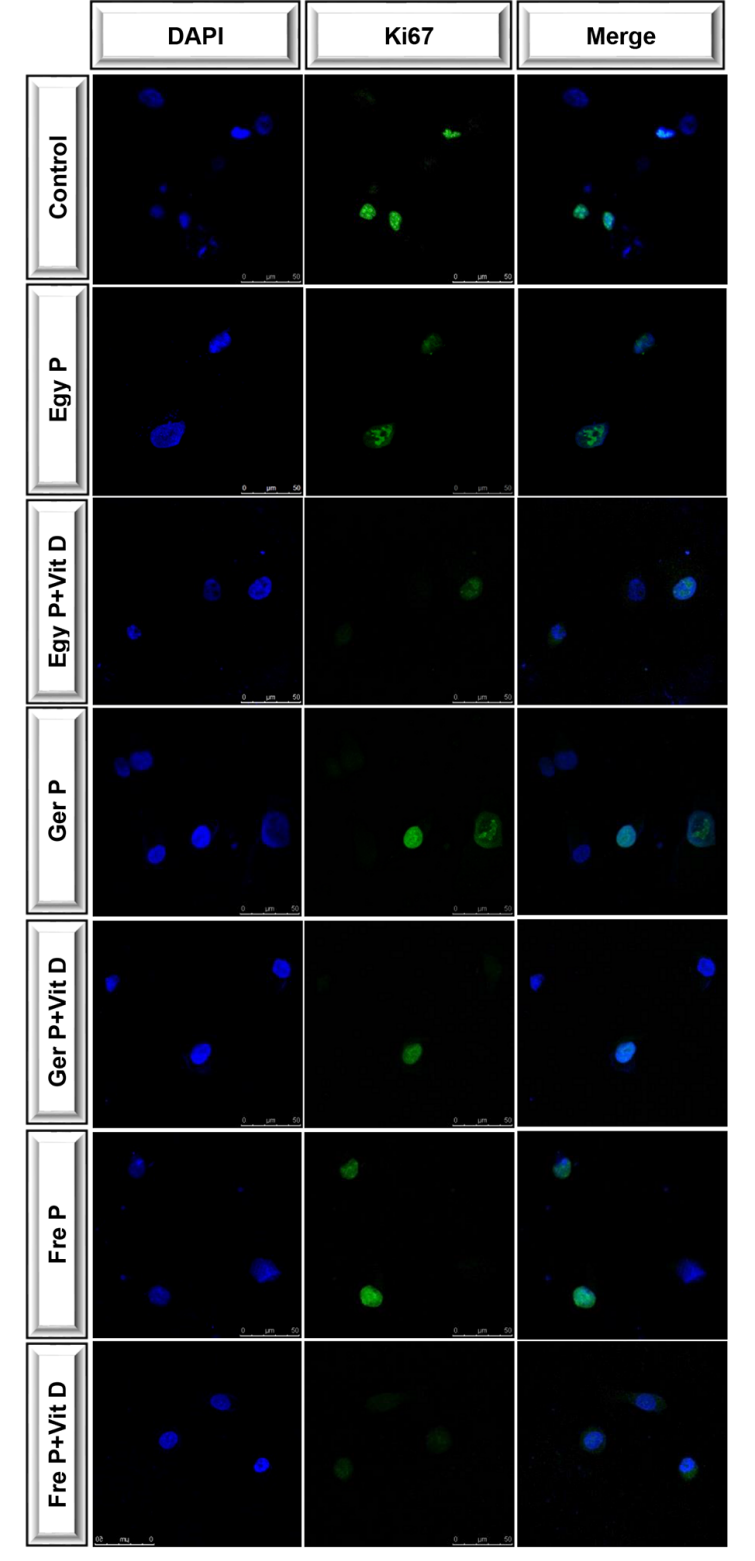
Immunofluorescence with the proliferative marker, Ki67. Human OVCAR4 cells were incubated for 48 h. with the IC50s of P from different geographical origin either alone or co-supplemented with vitamin D using the results obtained from Drug Reduction Indices analysis which were briefly as follow: 38.7045 μg/mL for Egy *P* + 0.01480 μg/mL for Vit D, 47.4519 μg/mL for Ger *P* + 0.01815 μg/mL for Vit D, and 36.9895 μg/mL for Fre *P* + 0.01415 μg/mL for Vit D. The left column was stained with DAPI to indicate the nuclei; the middle column was stained with Ki67 to indicate proliferation; and the right column contains the merged images. Scale bar: 50 μm

In an attempt to further clarify whether P treatment co-supplemented or not with Vit D affects the tumorigenicity of OVCAR4 cells, we investigated the expression of Ki67 which is a nuclear protein that is tightly linked to the cell cycle. Ki67 is associated with the proliferative activity of cell populations in malignant tumors, allowing it to be used as a marker for tumor progression [49]. In our experiment, the proliferative activity of OVCAR4 cells treated with P originating from different geographical regions is well manifested by Ki67 immnunofluorescent staining (Fig. 4). Co-supplementation with Vit D inhibits predominately the proliferative activity of cell population with the Fre P as manifested by Ki67 expression, while it reduces considerably Ki67-positive cells, particularly with the Ger P, followed by the Egy P.

## Discussion

Globally, ovarian cancer remains among the leading cause of death with regard to gynecological cancers. The standard treatment is cytoreductive surgery combined to chemotherapy. The response rate to first-line therapy is around 80–90%, but most patients relapse and develop chemotherapy resistance and the 5-year survival rate is < 30% [50].

Propolis is undoubtedly a naturally occurring extract with a complex chemical composition that contains a wide range of physiologically active phytochemicals, including flavonoids, terpenes, alcohols, phenolic acids, and their derivatives, which may have a variety of biological potentials [51, 52]. Propolis’ chemical composition is significantly influenced by plant origin, geography, climate, harvest times, and genetic variations across bee races [53–55]. In the current study, the propolis from different geographic regions including Egypt, Germany and France exerts anticancer properties against OVCAR4 cancer cells. The most active extract is from France then Egypt and Germany.

**Fig. 5:**
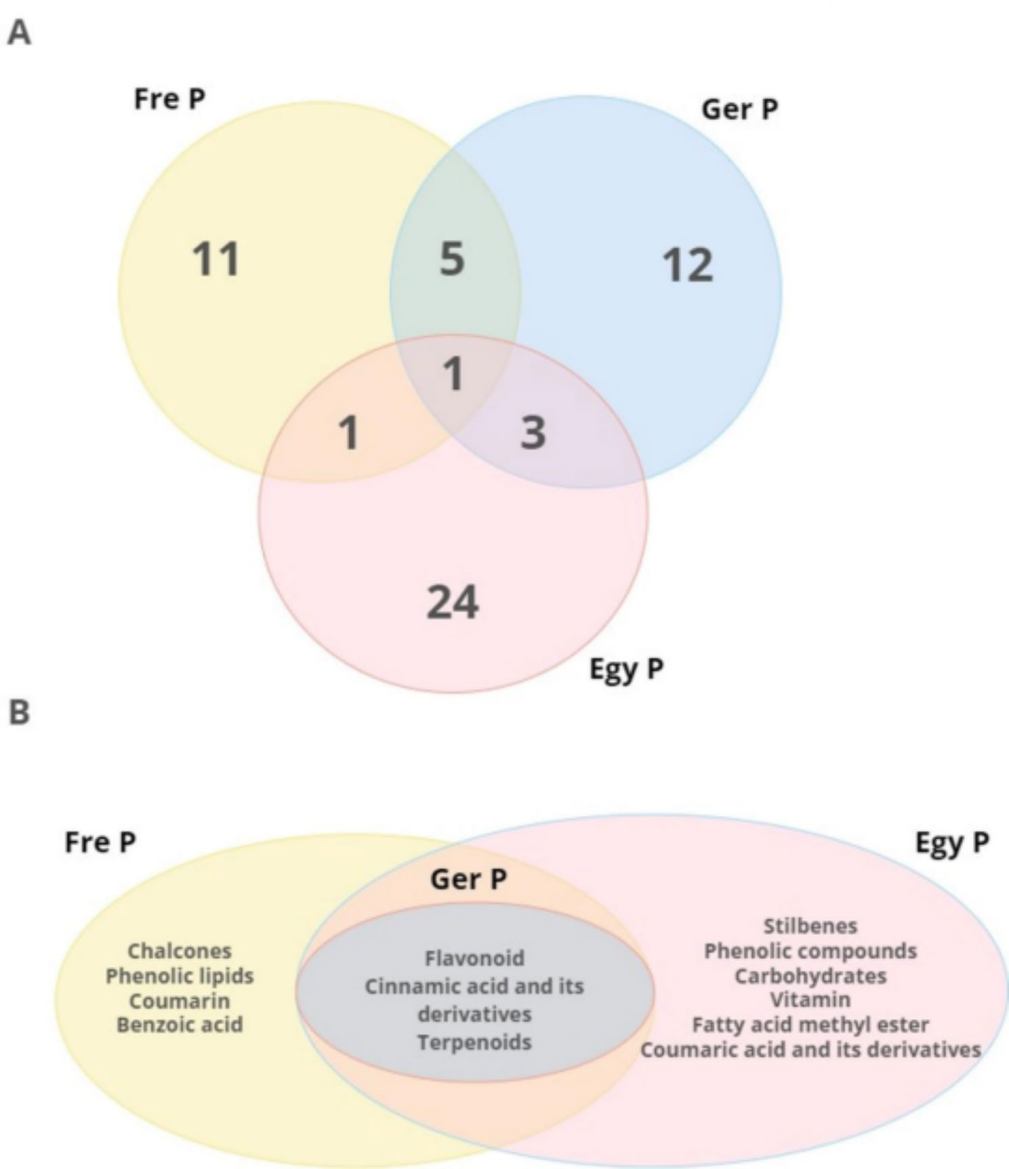
Venn diagram illustrating (**A**) The distribution of the number of specific and/or common metabolites in the different types of propolis analyzed, and (**B**) The different categories of specific metabolites identified by LC-MS/MS analysis in each type of propolis

Chemically propolis is composed of more than 180 different types of chemicals. As a result more than 300 different components have been previously identified in propolis collected from different regions. The percentage of diverse material present in propolis depends upon the time of its collection and also on the geographical origin [14, 56, 57]. Our LC-MS/MS analysis resulted in the identification of 57 compounds; 49 of which were actually found in propolis and are shown in Table 1. Each propolis sample was characterized by a number of specific and/or common metabolites (Fig. 5). The number of specific compounds were 11, 24, and 12 (Fig. 5A), classified into 7, 9, and 3 categories (Fig. 5B) for Fre P, Egy P, and Ger P, respectively. Even though flavonoid, cinnamic acid and its derivatives, and terpenoides are common in the different types of propolis, it is important to mention that the most abundant flavonoid is reported in Egy P including 13 metabolites, while the Ger P revealed plentiful of cinnamic acid and its derivatives including 5 compounds (Table S1). Artepillin C and caffeic acid, which were identified in our study only in the Fre P, are among the major anti-cancer ingredients of propolis^1^. The highest antiproliferative potential of Fre P may be therefore due to the presence of these two compounds. Artepillin C has been shown to exert direct antiproliferative, cytotoxic and apoptotic effects both in vitro on breast [58], colon [59] or lung cancer cells [1] and in vivo by inhibiting the growth of mice xenografts [60–62]. A recent study shows that artepillin C potently sensitized the resistant prostate cancer cells to treatment by inducing apoptotic cell death due to mitochondrial dysfunction [63]. Moreover, caffeic acid and its derivatives have been reported as potential modulators of oncogenic molecular pathways in a huge variety of cancer cells including, but not limited to, melanoma [64], colorectal [65], glioblastoma [66], osteosarcoma [67], and prostate cancer [68]. Similarities and variances in the rest of metabolites among the propolis samples originating from different regions were reported. Previous identification of pinocembrin, galangin, *p*-coumaric acid, and caffeic acid in Egy P, Ger P, and Fre P were noted [69–71]. Pinocembrin is a compound that was isolated from propolis and has anticancer properties on two different types of human colon cancer cells [72]. Galangin’s anti-cancer properties were shown. Human colon cancer cells were exposed to galangin, which caused apoptosis and DNA condensation in a dose-dependent way [73].

Propolis and its derivatives also have anticancer properties. The fundamental mechanisms underlying the development of cancer, including cell proliferation, evading apoptosis, angiogenesis, invasion, and metastasis, can be influenced by both propolis extracts and active chemicals. Propolis anticancer properties rely on its bioactive components, mainly flavonoid, phenolic, and aromatic component composition [70].

Vitamin D, traditionally known as an essential nutrient, is a precursor of a potent steroid hormone that regulates a broad spectrum of physiological processes. Accumulating data reported deficiency and/or dysregulated metabolism and functions of vitamin D in many types of cancer confirming thereby its antitumorigenic effects which are mainly best understood in colorectal, breast, and prostate cancer and much less in ovarian cancer [15, 74] In accordance with previous studies, our results demonstrated that the inhibitory effect of propolis on the proliferative activity of OVCAR4 cell population is augmented by vitamin D co-supplementation (Fre *P* > Ger *P* > Egy P). Such a synergism between propolis and vitamin D is confirmed by the decreased expression of ki67 which is a proliferative marker used routinely in the pathologic evaluation for all cancers. Moreover, in agreement with this finding, previous observational studies reported an inverse correlations of serum vitamin D with ki67 expression [75–77]. However, Lawler et al. observed no associations between serum vitamin D and ki67 marker expression in colorectal cancer patients, whereas an inverse association between vitamin D binding protein and tumor Ki67 explains the reduced mortality [78].

## Limitations

Although in vitro studies have massively promoted our understanding of mechanism of action of cancer progression and development, our work has some limitations that have to be taken into account: (i) We have tested the effects of the different propolis types on one kind only of human ovarian cancer cell line (OVCAR4). Comparaison between several kinds of ovarian cance cell line will be envisageable in future investigation, (ii) There are difficulties in simulating the consequences of long term treatment in vitro, and finally (iii) We cannot accurately replicate neither the inherent complexity of organ cells in living organisms nor the internal environment of human body.

## Conclusions

Ovarian cancer ranks among the deadliest gynecologic malignancy. Natural products were explored as an adjuvant treatment to improve therapeutic outcomes. LC–LTQ–MS/MS analysis of three different propolis originating from Egypt, Germany and France allowed for metabolites characterization as well as the investigation of compositional heterogeneity. Herein, we identified 57 compounds classified into 13 categories, of which flavonoid and cinnamic acid and its derivatives contain the most abundant metabolites. Fre P has shown the highest cell viability inhibition in human OVCAR4 ovarian cancer cells supplemented or not with vitamin D, followed by Egy P, and then, Ger P. This finding may be because the Fre P is the only type that contains artepillin C and caffeic acid which are among the major anti-cancer ingredients of propolis. Moreover, our results provided evidence for the differential antiproliferative efficacy in vitro of each propolis sample (Fre *P* > Ger *P* > Egy P) as manifested by Ki67 expression. Also, the demonstrated synergism between P and Vit D in the present investigation will permit further dose reduction of both drugs in future studies while preserving their anticarcinogenic effects. Altogether, propolis seems a valuable anticarcinogenic agent for further consideration. It has also the potential to upgrade ovarian cancer cells chemotherapeutic agents.

## Materials and methods

### Propolis samples

Liquid vitamin D (Art-Nr 54401) was purchased from Unimedic Pharma (Matfors, Sweden). Three different kinds of P were used in this study: raw Egy P was obtained from the Apiary of Department of Bee Research, Plant Protection Research Institute, Agricultural Research Agriculture Research Center at Dokki, Giza, Egypt. Ger P powder (Art-Nr 1334) was purchased from Aspermühle, Naturwaren-Niederrhein GmbH (Goch-Asperden, Germany), while Fre P powder (Ref. POUPROP40) was obtained from Propolia, Apimab laboratoires (Avenue du Lac, Clermont l’Hérault, France).

### Extraction of propolis

Egyptian propolis was extracted using ethanol. In brief, 5 g of the raw material was dissolved three times in 100 mL of ethanol. The suspension was filtered using Whatman filter paper (No. 1). The filtrate was evaporated to near-dryness using a rotary evaporator at low pressure. The propolis extract was stored in the refrigerator until used.

### Chemical analysis of propolis extracts

Extracts of propolis were analyzed using LC–MS/MS in positive ion modes. A Shimadzu LC-10 high performance liquid chromatography (HPLC) with a Grace Vydac Everest Narrowbore C_18_ column (100 mm × 2.1 mm i.d., 5 μm, 300 Å). LC-MS, connected to an LCQ electrospray ion trap MS (Thermo Finnigan, San Jose, CA) was utilized with a mass range of 200–5000 m/z. A 2 μL sample was injected using an autosampler. The solvents used were 95% H_2_O in formic acid (0.1%) (A) and 95% acetonitrile in formic acid (0.1%) (B). Gradient elution ranged from 5 to 95% solvent (B), then column conditioning to 5% solvent (B) at 300 μL/min flow rate. The elution time was 40 min.

Foundation 3.1 Xcalibur 3.1.6610 was used to analyze the data. Additionally, MS Convert from the ProteoWizard suite (http://proteowizard.sourceforge.net/tools.shtml; access on 12 November 2022) was used to convert the raw data files to mzXML format. GNPS online workflow was used to generate the molecular network [18, 19]. GNPS was generated for the positive ions using the following parameters: parent mass tolerance of 2 Da and an MS/MS fragment ion tolerance of 0.5 Da. The network’s spectra were then validated against the spectral libraries and literature data of GNPS. Cytoscape software was used to analyze and edit the molecular networks. The parent mass of each node served as a label. A pie slice proportionates to the number of MS/MS spectra for each parent mass and a color designating the source of the sample [79].

### Cell culture

Human OVCAR4 cells were maintained in Dulbecco’s Modified Eagle Medium (DMEM) with a low content of glucose (1 g/L) and supplemented with 10% fetal bovine serum (FBS), 1 mM glutamine, 1% antibiotics (penicillin-streptomycin) and 1 mM pyruvate at 37 °C under a humidified atmosphere containing 5% CO_2_. Cells were subcultured using a solution of 0.25% trypsine-0.25 mM ethylene-diaminetetraacetic acid (EDTA) and the medium was changed twice a week. Cell line was checked before the experiment to ensure it is mycoplasma-free. Each treatment group was made of 3 replicas of 3 independent experiments and maintained for 48 h.

### MTT assay

Cell viability was measured by MTT assays [80]. A total of 5 × 10^3^ cells/well was seeded onto a 96-well plate and incubated at 37 °C. After 24 h, the culture medium of each well was replaced with fresh medium and cells were treated with different concentrations (1–400 μg/mL), of P from different geographical sources, and/or Vit D (0.0015–0.15 μg/mL). In each experiment, complete growth medium without any treatment was also used as a control. Cells were incubated at 37 °C for 48 h. Then, cytotoxicity studies were done to determine via the survival curves the concentration of P with or without vitamin D co-supplementation that reaches 50% growth inhibition (IC_50_) using MTT assay. The media were therefore removed and 200 μL of MTT (3-(4,5-dimethylthiazol-2-yl)-2,5-diphenyltetrazolium bromide) solution at a final concentration of 1 mg/mL was added and left in darkness for 4 h, after which the MTT was removed and 100μL of dimethyl sulfoxide (DMSO) was added. Absorbance was measured in a microplate reader spectrophotometer at 560 nm. Each sample was tested in three independent sets with triplicate points. Values were expressed as the mean *±* SEM of at least three independent experiments and represented graphically.

### Analysis of the anti-proliferative effect of the drug combination

The hypothesized anti-tumour interaction between P and Vit D on OVCAR4 cells was evaluated using an MTT assay. Cells were incubated with P and/or Vit D using the same concentration ranges used in the former study for 48 h, and the cytotoxicity was assessed. To quantify the interaction synergism or antagonism, the combination index (CI) was determined as described by Chou [50], where CI < 1 indicates synergism, = 1 indicates additive action and > 1 indicates antagonistic effects. Moreover, the dose reduction index (DRI), expressed as the synergy of the combination of two drugs, was calculated as the fold-decrease in the dose of each drug independently related to their dose in combination using CompuSyn software, version 1 [53].

### Immunofluorescence Assay

Cells were grown on coverslips coated with poly-L-lysine and incubated for 48 h. with the IC_50_ concentrations of P from different geographical origin either alone or co-supplemented with vitamin D using the results obtained from Drug Reduction Indices (DRIs) analysis demonstrated in Table 2 which were briefly as follow: (47.4519 μg/mL for Ger *P* + 0.01815 μg/mL for Vit D, 38.7045 μg/mL for Egy *P* + 0.01480 μg/mL for Vit D, 36.9895 μg/mL for Fre *P* + 0.01415 μg/mL for Vit D.

Treated cells were then washed with PBS and fixed in 4% paraformaldehyde for 15 min. After washing 3 times with PBS for 5 min, the samples were air-dried, blocking solution was added to the slides and the cells were incubated for 5 min at room temperature, after which the blocking solution was drained away. The samples were incubated overnight with anti-Ki67 (proliferation marker), 100 μL/slide (1:500 dilution with PBS) at 4 °C. Cells were washed three times with PBS, 5 min each wash, and subsequently incubated for 1 h at room temperature with Alexa-Fluor-488 antibody. Cell nuclei were stained with 4,6-diamidino-2-phenylindole (DAPI) for 3 min at room temperature. Cells stained with DAPI were washed with PBS for 30 min, and fluorescence was observed using a Zeiss Axioplan 2 fluorescence microscope. Images were taken from five visible fields of interest from each immunocytochemical staining slides (×40). The ratio of stained positive nuclei to unstained negative ones was counted in randomly chosen 40× magnification fields.

### Statistical analysis

Data were expressed as the mean ± SEM. A one-way analysis of variance (ANOVA) followed by post hoc Tukey’s multiple comparison test was used to analyse multiple comparisons, and the differences were considered significant at *p* < 0.05. All statistical analyses and graphical data presentations were performed using Graph Pad Prism^®^ software package version 6 (GraphPad Software Inc., CA, USA).

## Electronic supplementary material

Below is the link to the electronic supplementary material.

Supplementary Material 1

## Data Availability

All data generated and analyzed in this study are included in this article.
