# Supplementary material for: Evaluation of the cytotoxic activity of chemically characterized propolis originating from different geographic regions and vitamin D co-supplementation against human ovarian cancer cells
Source: J Ovarian Res. 2024 Sep 7;17:181. doi: 10.1186/s13048-024-01500-6 (PMC11380329; doi:10.1186/s13048-024-01500-6)
Supplement: Supplementary file 1 — Supplementary Material 1 [file 13048_2024_1500_MOESM1_ESM.docx]

**Table S1**. Characterization of the different categories of specific metabolites identified using LC–LTQ–MS/MS analysis in each type of propolis originating from different regions.

| **Fre P** |
| --- |
| **Flavonoid** |
| 3’,4’-Di-*O*-benzyl-7-*O*-(2-hydroxyethyl)-3*-O*-methylquercetin |
| **Cinnamic acid and its derivatives** |
| Artepillin C (3,5-Diprenyl-4-hydroxycinnamic acid) |
| Caffeic acid |
| (2*E*)-3-[7-(3-methyl-2-buten-1-yl)-2-(1-methylethenyl)-5-benzofuranyl]-2-propenoic acid |
| 3-Prenyl-4-methoxy cinnamic acid |
| **Chalcones** |
| 2',6'-Dihydroxy-4'-methoxydihydrochalcone |
| (E,E,E)-4,2’,4’-Trihydroxy-3’-(7’’-hydroxy-3’’,7’’-dimethyloct-2’’,5’’-dienyl)-chalcone |
| **Terpenoids** |
| Acetylisocupressic acid |
| **Phenolic lipids** |
| 5-(12’*Z*-Heptadecenyl)-resorcinol |
| **Coumarin** |
| Esculetin |
| **Benzoic acid** |
| 4-Methoxybenzoic acid |
| **Egy P** |
| **Flavonoid** |
| 5-Hydroxy-4"-4"-dimethyl-5"-methyl-5"-H-dihrofurano(2",3"6,6) flavanone- |
| (-)-Liquiritigenin |
| 3’,5‑Dihydroxy‑4’,7‑dimenthoxy flavones |
| Galangin |
| 2’-Hydroxyformononetin |
| Medicarpin |
| 7-Hydroxy-8-methoxyflavanone |
| 7-Hydroxyflavanone |
| (*2R*,*3R*)-pinobanksin 3-(2-methyl)-butyrate |
| (-)-Mucronulatol |
| Kaempferide |
| Rhamnocitrin |
| 6-(1,1-dimethyl allyl) pinocembrin |
| **Cinnamic acid and its derivatives** |
| Chlorogenic acid |
| *p*-Coumaric acid |
| **Terpenoids** |
| Poilaneic acid |
| **Stilbenes** |
| 5,4’-Dihydroxy-3,3’-dimethoxy-2-prenyl-E-stilbene |
| **Phenolic compounds** |
| Obtusaquinol |
| (*Z*)-1-(2’-methoxy-4’,5’-dihydroxyphenyl)-2-(3-phenyl)propene |
| Trans-3,5-dihydroxy-1,7-diphenyl-hept-1-ene |
| **Carbohydrates** |
| Glycan 4.beta.-Galactobiose |
| **Vitamin** |
| Tocopherol |
| **Fatty acid methyl ester** |
| *cis*-7-Hexadecenoic acid methyl ester |
| **Coumaric acid and its derivatives** |
| 2-Acetyl-1,3-diferuloylglycerol |
| **Ger P** |
| **Flavonoid** |
| 5,7,3’,4’‑Tetrahydroxy‑6‑*C*‑geranylflavanone |
| (2*R*,3*R*)-6-[1-(4-Hydroxy-3-methoxyphenyl)prop-2-en-1-yl]pinobanksin |
| Daidzein |
| Hesperitin5,7‑dimethyl ether |
| Pinostrobin |
| Schweinfurthin A |
| **Cinnamic acid and its derivatives** |
| Dihydrocaffeic acid |
| Cinnamyl caffeate |
| Caffeoyl coumaroyl acetyl glycerol |
| Dimethoxycinnamic acid |
| (2*E*)-3-(2,2-dimethyl-2H-1-benzopyran-6-yl)-2-propenoic acid |
| **Terpenoids** |
| Mangiferonic acid |
